# Structure of a Complex Phosphoglycan Epitope from gp72 of *Trypanosoma cruzi* *

**DOI:** 10.1074/jbc.M113.452763

**Published:** 2013-02-22

**Authors:** Simon Allen, Julia M. Richardson, Angela Mehlert, Michael A. J. Ferguson

**Affiliations:** From the ‡ Division of Biological Chemistry and Drug Discovery, College of Life Sciences, University of Dundee, Dundee DD1 5EH, Scotland, United Kingdom and; the § School of Biological Sciences, University of Edinburgh, Edinburgh EH9 3JR, Scotland, United Kingdom

**Keywords:** Carbohydrate Glycoprotein, Carbohydrate Structure, Glycoprotein Structure, Glycosylation, Parasitology, Trypanosoma cruzi, Carbohydrate Epitope, Galactofuranose, gp72, Phosphosaccharide

## Abstract

The parasitic protozoan organism *Trypanosoma cruzi* is the causative agent of Chagas disease. The insect vector-dwelling epimastigote form of the organism expresses a low abundance glycoprotein associated with the flagellum adhesion zone, called gp72. The gp72 glycoprotein was first identified with an anti-carbohydrate IgG3 monoclonal antibody called WIC29.26 and has been shown to have an unusual sugar composition. Here, we describe a new way to isolate the WIC29.26 carbohydrate epitope of gp72. Using ^1^H NMR and mass spectrometry before and after derivatization, we provide an almost complete primary chemical structure for the epitope, which is that of a complex phosphosaccharide: Gal*f*β1–4Rha*p*α1–2Fuc*p*α1-4(Galpβ1–3)(Galpα1–2)Xyl*p*β1–4Xyl*p*β1–3(Xyl*p*β1–2Gal*p*α1-4(Gal*p*β1–3)(Rha*p*α1–2)Fuc*p*α1–4)GlcNAc*p*, with phosphate attached to one or other of the two Gal*p* terminal residues and in which all residues are of the d-absolute configuration, except for fucose and rhamnose which are l. Combined with previous data (Haynes, P. A., Ferguson, M. A., and Cross, G. A. (1996) *Glycobiology* 6, 869–878), we postulate that this complex structure and its variants lacking one or more residues are linked to Thr and Ser residues in gp72 via a phosphodiester linkage (GlcNAc*p*α1-*P*-Thr/Ser) and that these units may form phosphosaccharide repeats through GlcNAc*p*α1-*P*-Gal*p* linkages. The gp72 glycoprotein is associated with the flagellum adhesion zone on the parasite surface, and its ligation has been implicated in inhibiting parasite differentiation from the epimastigote to the metacyclic trypomastigote stage. The detailed structure of the unique phosphosaccharide component of gp72 reported here provides a template for future biosynthetic and functional studies.

## Teaser

**Background:** *Trypanosoma cruzi* expresses a highly immunogenic carbohydrate epitope in glycoprotein gp72.

**Results:** The epitope structure was solved using NMR and mass spectrometry and shown to be a phosphosaccharide, containing l-rhamnopyranose, l-fucopyranose, d-galactopyranose, d-galactofuranose, d-xylopyranose, and *N*-acetylglucosamine.

**Conclusion:** This is one of the most complex eukaryotic protein-linked carbohydrate structures yet described.

**Significance:** gp72 has been implicated in parasite differentiation, flagellar adhesion, and insect infectivity.

## Introduction

The parasitic protozoan organism *Trypanosoma cruzi* is transmitted by biting insect (reduviid bug) vectors. In man, transmission can also occur through blood transfusion. *T. cruzi* infection can lead to Chagas disease, characterized by an acute phase involving fever, malaise, facial edema, lymphadenopathy, and hepatosplenomegaly (fatal in about 5% of childhood cases) followed by a chronic phase of indeterminate length that can lead to serious and life-threatening sequelae such as dilated cardiomyopathy, megaesophagus, and megacolon. Chagas disease was originally localized to South and Central America and the Southern United States through the natural range of the insect vectors, but worldwide migration has seen significant numbers of cases appear elsewhere in, for example, North America and Europe. Current estimates suggest more than 8 million people are infected, that 10–30% of these people are likely to develop serious medical consequences within 10–15 years of infection, and that the disease causes about 12,000 deaths annually. The current therapeutics, benznidazole and nifurtimox, have serious toxicity and efficacy issues, and the development of new therapeutics for Chagas disease is a major priority for the World Health Organization and the Drugs for Neglected Diseases initiative.

The organism undergoes a complex life cycle between the insect vector and the mammalian host. It replicates as the epimastigote form in the midgut of the reduviid bug and, on migration to the hindgut, differentiates into the nonreplicating infectious metacyclic trypomastigote form that initiates host infection through insect fecal contamination of the bite wounds or of the eye orbit or mucous membranes of the mammalian host. The metacyclic trypomastigotes can invade a wide range of nucleated host cells where they escape the surrounding host parasitophorous vacuole membrane, differentiate into replicating amastigote forms, and divide in the host cell cytoplasm. Some amastigotes differentiate into nondividing bloodstream trypomastigote forms that are liberated when the infected host cells rupture. The bloodstream form trypomastigotes are responsible for infecting additional host cells and also for transmission to the reduviid bug vectors through bloodmeals. Once ingested by the vector, the bloodstream trypomastigote forms differentiate into dividing epimastigote forms, and the life cycle is completed.

The cell surface of *T. cruzi* is dominated by glycosylphosphatidylinositol-anchored mucin-like molecules and free glycosylphosphatidylinositol glycolipids. The structure and function of these surface molecules, and the trans-sialidases that add sialic acid to the mucin *O-*glycans, have been reviewed (1–5). In addition to these major surface components, some less abundant surface glycoproteins have been characterized, for example, the highly modified 13-amino acid NETNES glycoprotein that contains a glycosylphosphatidylinositol anchor, up to two conventional Man_5_GlcNAc_2_ *N*-linked glycans, and two tri-α-mannosyl glycans phosphate-linked (P-linked)^3^ to Ser residues (6). Complex P-linked glycans are also found in the proteophosphoglycans of the leishmania (7) and the Glutamic acid/Alanine Rich Protein of *Trypanosoma congolense* (8). However, P-linked glycans are relatively rare (9), and *Dictyostelium discoidium* is the only other organism where they have been described (10). The only other reported example of P-linked glycoprotein glycans in a trypanosomatid is for the gp72 glycoprotein of *T. cruzi* (11).

The glycoprotein gp72 was discovered when mAbs were first raised to whole *T. cruzi* epimastigotes. One of the highest titer antibodies (WIC29.26) affinity-purified a broad 72-kDa band from epimastigote detergent lysates (12) that proved to be a glycoprotein with very high carbohydrate (50%) content (13, 14). Early structural studies suggested that its novel glycans contained the WIC29.26 epitope and that they could be liberated by base treatment, suggesting linkage to Thr and/or Ser residues (13). The gene encoding gp72 was eventually cloned (15), revealing a hydroxyamino acid and proline-rich domain likely to carry the WIC29.26-reactive glycans, and subsequent structural studies suggested phosphodiester linkages between Thr and Ser residues and the glycan component (11). A partial structure was also suggested for the glycan component of: Gal*f-*dHex-dHex-(Gal*f*-)(*P*Gal*f*-)Xyl-Xyl-OH (11).^4^ The gp72 glycoprotein localizes to the flagellar adhesion zone (16) and plays a crucial role in flagellar adhesion (17) and parasite virulence (18, 19). Ligation of gp72 with the mAb WIC29.26 also prevents the differentiation of epimastigote form *T. cruzi* cells to metacyclic trypomastigotes (20), and it is thought that a similar interaction between a vector lectin and gp72 might serve to maintain midgut epimastigote infections. For all of these reasons, the structures of unusual glycans carried by gp72 are of interest. In this paper, we significantly revise and refine this partial structure.

## EXPERIMENTAL PROCEDURES

### 

#### 

##### Selection of a T. cruzi Strain Expressing the WIC29.26 Epitope

Continuous cultures of epimastigote cells from three strains of type I *T. cruzi* (Tulahuen, Silvio X10-4, and Silvio X10-7) and two strains of type II *T. cruzi* (CanIII and CanY) were grown in plastic culture flasks (25 cm^2^; Greiner) at 28 °C in liver-infused tryptose medium (70 mm sodium chloride, 5 mm potassium chloride, 20 mm disodium hydrogen phosphate, 10 mm glucose, 5 g/liter tryptose (Difco), 5 g/liter liver infusion broth (Difco), 10 mg/liter hemein (Sigma), adjusted to pH 7.0 with hydrochloric acid) supplemented with 10% heat-inactivated FCS. The cells were harvested by centrifugation and washed three times with PBS followed by hypotonic lysis in water (1 × 10^9^ cells/ml) with three freeze (liquid nitrogen)/thaw (50 °C) cycles. Lysates equivalent to 1 × 10^7^ cells were loaded and separated by SDS-PAGE along with molecular weight standards (broad range; Bio-Rad). Western blotting was performed using a Hoefer Semiphor semidry blotter onto nitrocellulose paper (Hybond-C; GE Healthcare), and protein transfer was confirmed by staining with Ponceau S or Amido Black. Immunodetection was carried out after blocking the membrane overnight with PBS, 0.1% Tween 20 (PBS-Tween) with 0.1% BSA and subsequently three washes with PBS-Tween. The membrane was incubated for 1 h with WIC29.26 mAb ascites fluid diluted 1 in 1000 with PBS-Tween, followed by three washes with PBS-Tween. The secondary biotinylated goat anti-mouse antibody, diluted 1 in 2000 in PBS-Tween, was incubated with the membrane for 30 min, followed by three PBS-Tween washes, and finally the membrane was incubated with extravidin-horeradish peroxidase diluted 1 in 1000 in PBS-Tween, for 30 min, followed by three PBS-Tween washes. The blot was then visualized using the ECL chemiluminescence kit (GE Healthcare) and exposure onto photographic film (Kodak X-OMAT-AR-5).

##### Purification of WIC29.26 Epitope-containing Peptides

Batch cultures (500 ml) of *T. cruzi* Can III strain were grown in liver-infused tryptose medium in polycarbonate Fernbach flasks (Nalgene) with nonabsorbent cotton wool seals at 28 °C on a flatbed orbital shaking incubator (New Brunswick Scientific) until they reached late log phase (∼5 × 10^7^ cells/ml). The cells were washed in PBS and hypotonically lysed, as outlined above, before dilution to 4 × 10^8^ cell equivalents/ml with PBS. Diluted lysates were then digested with Pronase^TM^ (1 mg for every 10^10^ cell equivalents) for 48 h at 37 °C (two drops of toluene were added as bacteriostatic agent). After digestion a fresh mixture of proteinase inhibitors were added to the following final concentrations: 0.8 mm phenymethylsulfonyl fluoride, 4.3 μm leupeptin, 0.2 mm *N*^α^-*p*-tosyl-l-lysine chloromethylketone, 0.8 mm 4-amidinophenylmethanesulfonyl fluoride, 1 mm iodoacetamide, 1 mm *N*-ethylmaleimide, 0.8 mm benzamide, 0.3 μm aprotinin, 3 mm EDTA, and 1 mm iodoacetic acid. The Pronase^TM^-digested lysates were then centrifuged at 100,000 × *g* for 1 h at 20 °C. The WIC29.26 epitope-containing glycopeptides were extracted from the digested lysates using a WIC29.26-Sepharose CL-4B column (made by coupling protein A affinity-purified WIC29.26 mAb to cyanogen bromide-activated Sepharose CL-4B beads (GE Healthcare) at 1 mg antibody per ml of gel, according to the manufacturer's instructions). The lysates were loaded onto the column at 10 ml/h, and then the column was washed thoroughly (until absorption at 280 nm was >0.01 absorbance units) with 0.1 m ammonium bicarbonate, 0.5 m sodium chloride, followed by 0.1 m ammonium bicarbonate and finally water. The WIC29.26 epitope-containing peptides were then eluted using 50 mm diethylamine. The eluted fractions were immediately lyophilized, followed by two further lyophilizations from water, to ensure complete removal of the diethylamine. The fractions were then reconstituted in water and dot blotted to localize the gp72. Briefly, aliquots of 0.4% of each fraction were applied to nitrocellulose paper, dried, then blocked and treated as described above for Western blotting. Fractions that gave positive reaction with the WIC29.26 mAb were pooled and frozen.

##### Monosaccharide Composition Analysis

Monosaccharide composition was determined by GC-MS of trimethylsilyl-derivitized methylglycosides prepared as described by Ferguson *et al.* (21). Aliquots of the resulting derivatives were analyzed by GC-MS (Agilent) using an EconoCap SE-54 column (30 m × 0.25 mm; AllTech) or a HP-5 column (30 m × 0.25 mm; Agilent) with helium as a carrier gas (0.5 ml/min). The GC splitless injector and MS-interface were held at 280 °C. The oven temperature gradient was as follows: 80 °C for 2 min, followed by 30 °C/min to 150 °C, 5 °C/min to 280 °C, 15 °C/min to 300 °C, hold at 300 °C for 20 min. Electron impact mass spectra were collected using selected ion monitoring time windows, and ion *m*/*z* values were as follows: 7.5–15.5 min, *m*/*z*: 214/217/272 (deoxyhexoses, pentoses and anhydro-hexitols); 15.5–20 min, *m*/*z*: 133/204/217 (hexoses); 20–23 min, *m*/*z*: 173/205/305/318 (scyllo-inositol and *N*-acetylhexosamines); 23 min to end, *m*/*z*: 204/217/298 (sialic acids). Quantification was based on empirically determined response factors from monosaccharide standard mixtures (equimolar mix of rhamnose, fucose, xylose, xylitol, mannose, galactose, glucose, galacticol, and *scyllo*-inositol) and integration of the selected ion chromatograms.

##### Determination of Absolute Configuration of Monosaccharides

The absolute (d- or l-) configuration of the monosaccharide components was determined by (+)-butanolysis (22). The (+)-2-butanolic-HCl reagent was prepared by adding 175 μl of acetyl chloride (Alltech) dropwise to 2.5 ml of (*S*)-(+)-2-butanol (99%, Sigma). (+)-2-Butyl-glycosides were formed using the method described above for methyl-glycosides but replacing the methanolic-HCl with (+)-2-butanolic-HCl. The (+)-2-butyl-glycosides were trimethylsilyl-derivatized and analyzed by GC-MS as above, except that linear ion scanning (*m*/*z* 240–650) was used instead of selected ion monitoring. Assignments of absolute configuration were made by comparison of retention times and peak patterns of authentic d- and l-monosaccharide standards.

##### The Release and Purification of Oligosaccharides from WIC29.26 Affinity-purified Glycopeptides

The samples were incubated with 48% aqueous hydrofluoric acid (aq HF) for 8 h at 0 °C. The HF was then removed by lyophilization followed by drying three times from water to ensure that all traces of HF were removed. Released oligosaccharides were applied to a Superdex Peptide HR10/30 gel filtration column (24 ml of bed volume; GE Healthcare). The column was held at 50 °C and eluted with 100 mm ammonium acetate through an in-line refractive index monitor. The fractions were lyophilized and then dried three times from water to remove residual ammonium acetate. Aliquots (4%) of each fraction were spotted onto a silica HPTLC plate and stained with orcinol reagent; orcinol reagent was prepared by dissolving 180 mg of orcinol in 5 ml of water and 75 ml of ethanol, acidifying on ice by dropwise addition of 10 ml of sulfuric acid, and storing in the dark at 4 °C. The orcinol reagent was applied to the HPTLC plate in a fume hood as a fine spray and allowed to dry prior to development at 100 °C for 0.5–2 min, depending on the amount of carbohydrate present.

##### NMR Spectroscopy

Aqueous HF-treated and gel filtration-purified samples were prepared for NMR by desalting on a mixed bed Chelex-100 (200–400 mesh; Bio-Rad) over Dowex AG-50W-X12 (200–400 mesh, hydrogen form; Bio-Rad) column to remove mono- and divalent cations. The sample was then dried three times from 99.9% D_2_O prior to reconstitution in 300 μl of 99.9% D_2_O and transfer to a Shigemi NMR tube. The samples were analyzed by ^1^H one- and two-dimensional NMR spectroscopy using a 600-MHz Varian INOVA NMR spectrometer and an 800-MHz Bruker DRX spectrometer. Spectra were recorded at a probe temperature of 310 K. Two-dimensional homonuclear ^1^H correlation spectroscopy (COSY), total correlation spectroscopy (TOCSY), and rotating frame nuclear Overhauser effect spectroscopy (ROESY) were performed at both 600 and 800 MHz using the parameters described in Table 1.

**TABLE 1:** **NMR spectrometer parameters used in NMR experiments**

Experiment	Field	SW*^a^*	Offset*^b^*	No. of experiments	No. of scans/experiment	Mixing time
	*MHz*	*ppm (Hz)*	*ppm*			*ms*
COSY	600	5.32 (3193)	3.65	1024	32	
TOCSY	600	5.32 (3193)	3.65	512	32	180
ROESY	600	8.33 (5000)	4.67	1024	56	350
TOCSY	800	8.75 (7002)	4.67	1024	24	180
ROESY	800	11.24 (8992)	5.91	1024	16	350

*^a^* SW, spectral width in both dimensions.

*^b^* Transmitter offset.

##### Methylation Linkage Analysis

Permethylated alditol acetates (PMAAs) for methylation linkage analysis were prepared by a protocol adapted from that described in Ref. 21. Briefly, samples were dried in 2-ml Reacti-Vials (Thermo), then dissolved in 100 μl of 10 mm triethylamine, and redried to convert phosphate groups to triethylamine salts. The samples were then dried twice from methanol to ensure dehydration. Subsequently, the samples were dissolved in 50 μl of Me_2_SO with mixing for 20 min. To this 50 μl, a 120 mg/ml fine suspension of NaOH in dry Me_2_SO was added and mixed for 20 min. Two aliquots of 10 μl and one of 20 μl of methyl iodide were added 10 min apart, and after mixing for a total of 50 min, 1 ml of water and 250 μl of chloroform were added, and the samples were vortexed for 1 min. The permethylated oligosaccharides were recovered in the chloroform phase and were transferred to a clean glass tube. The chloroform phase was washed with 2 ml of 100 mg/ml sodium thiosulfate (to remove free I_2_) followed by four 2-ml water washes. The chloroform was removed under nitrogen prior to hydrolysis of the permethylated oligosaccharides with 100 μl of 4 m trifluoroacetic acid at 100 °C for 4 h. The samples were then dried in a SpeedVac followed by drying twice more from water. The samples were reduced with 100 μl of freshly prepared 0.25 m sodium borodeuteride for 3 h at room temperature, with excess borodeuteride destroyed with 50 μl of acetic acid. The samples were dried and redried from four additions of 200 μl of methanol (to remove boric acid) and two of 10 μl of toluene (to remove residual acetic acid). The samples were then acetylated with 50 μl of acetic anhydride at 100 °C for 2.5 h prior to drying twice from 10 μl of toluene. Finally, the samples were dissolved in 250 μl of dichloromethane and extracted four times with 0.5 ml of water. The dichloromethane phase was dried under nitrogen and reconstituted in 50 μl of dichloromethane. Aliquots (1 μl) of the PMAA solution were then analyzed by GC-MS using an EconoCap SE-54 column (Alltech, 30 m × 0.25 mm) or an EconoCap EC-WAX column with helium carrier gas at 0.5 ml/min. The temperature program used was as described above. Electron impact mass spectra were collected using linear scanning over *m*/*z* 40–650; PMAAs were identified by comparison of the retention times and spectra of partially permethylated alditol acetate standards.

##### Electrospray Mass Spectrometry

The samples were dissolved at 10–20 pmol/μl in 50% acetonitrile, 0.2% formic acid for positive ion mode electrospray mass spectrometry or in 50% acetonitrile, 3 mm ammonium acetate to collect spectra in negative ion mode. The samples were loaded into nanospray tips (type F; Waters) for introduction into the electrospray source of a Q-TOF2 mass spectrometer (Waters) or Orbitrap XL mass spectrometer (Thermo). The capillary voltages were typically 0.9–1.1 kV.

## RESULTS

### 

#### 

##### Selection of a T. cruzi Strain Expressing the WIC29.26 Epitope

**FIGURE 1.**
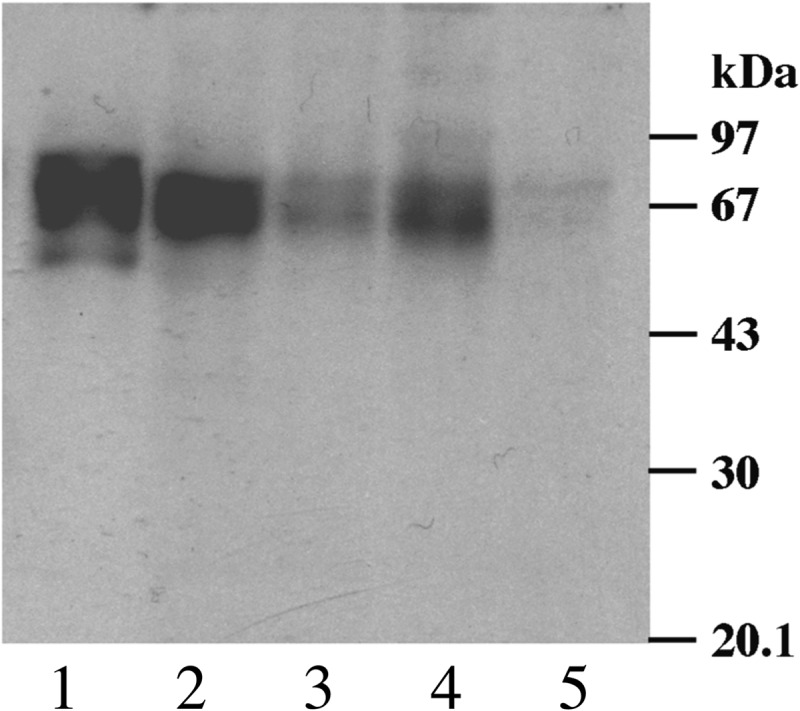
**Western blot of *T. cruzi* strains with mouse mAb WIC29.26.** *T. cruzi* lysates equivalent to 1 × 10^7^ cells of strains Can III (*lane 1*), Tulahuen (*lane 2*), Silvio X10-4 (*lane 3*), Silvio X10-^7^ (*lane 4*), and Y (*lane 5*) were separated on a 10% SDS-PAGE gel, transferred to nitrocellulose, and blotted with WIC29.26, followed by anti-mouse antibody/biotin conjugate and detected with extravidin-HRP. The positions of the molecular mass markers are shown on the *right*. The CanIII strain was selected for this study on the basis of the strongest immunoreactivity with mAb WIC29.26.

Previous structural studies on gp72 and the WIC29.26 epitope have used the Y strain of *T. cruzi* (11–14). However, before embarking on a detailed structural analysis of the epitope, we screened whole cell lysates of five strains of *T. cruzi* by Western blot with mAb WIC29.26. Based on this analysis (Fig. 1), we selected the strain CanIII for the large scale purification of glycopeptides from gp72, which appeared as a broad band with an apparent weight between 62 and 92 kDa carrying the WIC29.26 epitope (Fig. 1, *lane 1*).

##### Purification of WIC29.26 Epitope-containing Glycopeptides

Previous purifications of WIC29.26 epitope-containing glycoproteins have used *T. cruzi* detergent lysates as the starting material (11–14). We decided to avoid the use of detergents, because they are often incompatible with mass spectrometry and difficult to remove. The new purification protocol used here involved hypotonic lysis, freeze-thawing, and sonication to yield a fine suspension of broken cells. The suspension was then treated with Pronase, to liberate water-soluble glycopeptides, and centrifuged to remove insoluble material. The soluble material was applied to a WIC29.26 mAb affinity column, and after extensive washing, the WIC29.26 epitope-containing glycopeptides were eluted with 50 mm diethylamine. Fractions that reacted with WIC29.26 mAb by dot-blot were pooled. Monosaccharide composition analyses by GC-MS showed the presence of Rha, Fuc, Xyl, Gal, and GlcNAc (Table 2), characteristic of the WIC29.26 epitope and gp72 (13, 14) and variable amounts of Glc. The latter sugar is a common contaminant in glycoconjugate preparations and was found later not to be a component of the WIC29.26 epitope.

**TABLE 2:** **Monosaccharide composition of the WIC29.26 epitope**

Sugar	Affinity-purified glycopeptides*^a^*		Aq. HF liberated oligosaccharides*^a^*	
	This study	Previous data*^b^*	This study	Previous data*^c^*
Rha	0.8	0.9	0.7	0.8
Fuc	1.0	1.0	1.0	1.0
Xyl	2.5	2.0	2.0	1.4
Gal	1.9	2.9	1.8	2.7
Glc	1.0	0.5	0.5	0.0
GlcNAc	+*^d^*	NR*^e^*	+*^d^*	NR*^e^*

*^a^* The values are expressed as molar ratios, with fucose arbitrarily set to 1.0.

*^b^* Values taken from Ref. 11.

*^c^* Values taken from Ref. 11. The values are for the acid-released and purified MAL-P2 fraction described in that paper.

*^d^* Detected but not quantitated.

*^e^* NR, not reported.

##### Determination of the Absolute Configuration of the Sugars in the WIC29.26 Epitope-containing Glycopeptides

There is precedent for unusual monosaccharides in trypanosomatid glycoconjugates. For example, the lipophosphoglycan of *Leishmania major* (24, 25) and the lipoarabinogalactan of *Crithidia fasciculata* (26, 27) contain d-Ara*p* rather than l-Ara*p*. We therefore determined the absolute configuration (d or l) of the neutral monosaccharides in the WIC29.26 epitope-containing glycopeptides using the method in Ref. 22. This method uses an acidified chiral alcohol ((+)-2-butanol) to make diastereoisomer alkyl-glycosides of the monosaccharide constituents that are subsequently resolved as their trimethylsilyl-derivatives by conventional capillary GC-MS. By comparison with the retention times of (+)-2-butyl-glycoside-trimethylsilyl-derivatives from the sample with those of authentic d-Fuc, d-Xyl, d-Gal, d-Glc, l-Fuc, l-Xyl, l-Gal, l-Glc, and l-Rha sugar standards, the glycopeptide sugars were unambiguously identified as l-Fuc, d-Xyl, d-Gal, and d-Glc (data not shown). The glycopeptide sample Rha derivatives co-chromatographed exactly with those of the authentic l-Rha standard, suggesting that the sample sugar is indeed l-Rha. However, because an authentic standard of d-Rha was not available for comparison, this assignment is necessarily incomplete. The analysis did not allow the assignment of the absolute configuration of GlcNAc, which is assumed, from the presence of conventional eukaryotic hexosamine pathway genes in *T. cruzi* (27), to be d-GlcNAc.

##### The Release and Purification of Oligosaccharides from WIC29.26 Affinity-purified Glycopeptides

Mild acid treatment has been used previously to release phosphoglycans from WIC29.26 affinity-purified glycopeptides (11). The methodology is based on the mild acid treatment used to degrade the phosphoglycan chain of lipophosphoglycan from *Leishmania* into individual phosphoglycan units (23). The drawbacks of this approach are that the conditions are difficult to control, because the low concentrations of acid (40 mm trifluoroacetic acid) used are easily buffered by contaminating salts in the sample, and the constituent galactofuranose and deoxyhexoses within the structure are also rather labile to mild acid. For these reasons, we chose to use partial aqueous HF dephosphorylation as an alternative means to break the glycopeptides down into analysable fragments. We selected 48% aq. HF for 8 h at 0 °C for this procedure because preliminary experiments using synthetic standards of (–PO_3_H-6Galβ1–4Manα1-*O*-)_10_ and Galfβ1–3Manα1-*O*-(CH_2_)_8_-COOH suggested that these conditions substantially cleaved the Manα1-PO_4_H-6Gal phosphodiester bonds while releasing only 10% of the Gal*f* from Gal*f*β1–3Manα1-*O*-(CH_2_)_8_-COOH (data not shown).

Glycopeptides, affinity-purified on WIC29.26-immobilized antibody from ∼2.4 × 10^12^ cells, were treated with 48% aq. HF for 8 h at 0 °C, and the reagent was removed by freeze-drying. The products were applied to a Superdex Peptide HR10/30 gel filtration column. Aliquots of the fractions were dotted onto a silica HPTLC plate and stained for carbohydrate with orcinol reagent. The orcinol stain coincided with a major refractive index detector peak, and these fractions were pooled. Monosaccharide composition analysis of this fraction revealed a composition similar to the starting material but with significantly less contaminating glucose (Table 2).

##### NMR Analysis of the aq. HF-released Glycans

**FIGURE 2.**
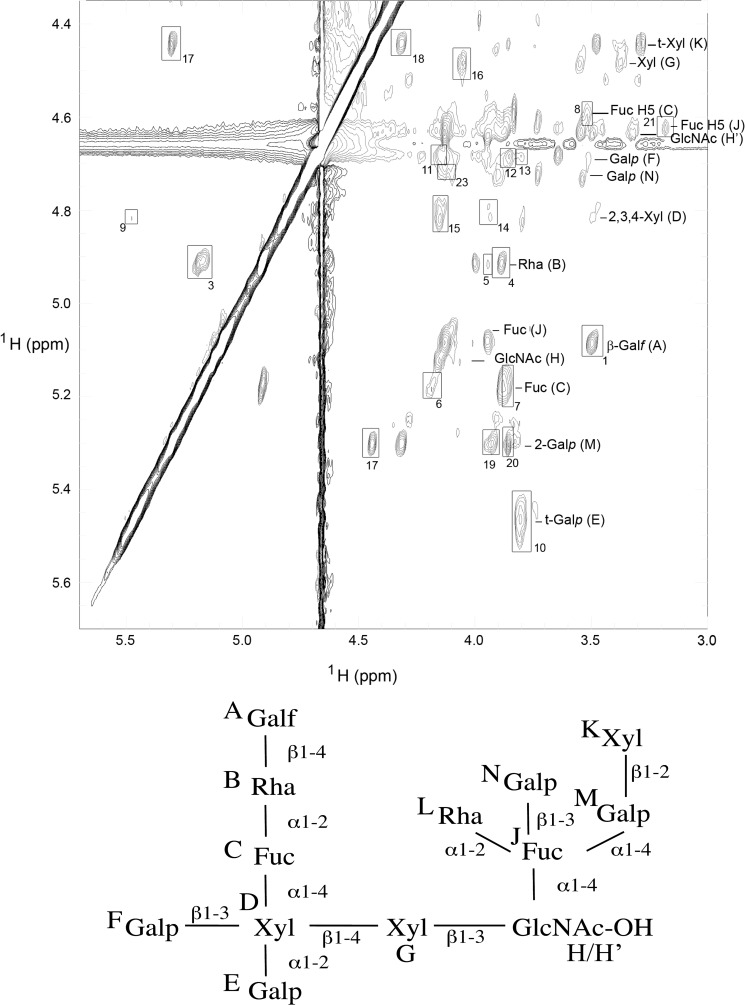
**NMR analysis of the WIC29.26 carbohydrate epitope.** The anomeric region of the two-dimensional ^1^H ROESY spectrum is shown. Monosaccharide spin systems are labeled, and the ROEs are numbered as in Table 2. The residue descriptors (*A–M*) are shown below the spectrum on the final deduced structure, for convenience.

The aq. HF-released and gel filtration purified glycan fraction was desalted, exchanged into D_2_O, and analyzed by one- and two-dimensional (TOCSY, ROESY, and COSY) ^1^H NMR. The NMR data indicated that the sample was heterogeneous, and ES-MS confirmed this (see below). Nevertheless, the constituent monosaccharide spin systems were identified by COSY and TOCSY (Table 3), and a principal inter-residue connectivity network was identified from the ROEs in the ROESY spectrum (Fig. 2 and Table 4). This involves ROEs from residue A-H1 to residue B-H4 and Me, from residue B-H1 to residue C-H1 and H2 and from residue C-H1 to residue D-H4 and H5e; consistent with the sequence Gal*f*β1–4Rha*p*α1–2Fuc*p*α1–4Xyl*p*. Five more ROEs involving residue D were apparent: one from residue E-H1 to residue D-H2 (corresponding to Gal*p*α1–2Xyl*p*); three from residue F-H1 to residue D-H2, H3, and H4 (corresponding to Gal*p*β1–3Xyl*p*); and one from residue D-H1 to residue G-H4 (corresponding to Xyl*p*β1–4 Xyl*p*). Chemical shift data indicate that residues D and G experience two chemical environments; this is most likely due to the attachment of residue G to residue H (as evidenced by an ROE from residue G-H1 to residue H-H3), where residue H exists in two forms, H and H′. Chemical shift data suggest that H and H′ are the α and β anomers of a free reducing GlcNAc residue. Thus, residues D-G-H/H′ correspond to Xylβ1–4Xylβ1–3GlcNAc. Methylation linkage analysis (see below) indicates a 3,4-di-*O*-substituted GlcNAc reducing terminus. An ROE from residue J-H5 to G-H2 indicates that residue J (αFuc) and G are close in space, possibly because of both being connected to GlcNAc. Analysis of a model compound, Lewis-a Galβ1–3(Fucα1–4)GlcNAc (data not shown) showed a Fuc H-5 to Gal H-2 ROE, indicating that the J-H5 to G-H2 ROE seen in the *T. cruzi* glycan spectra is consistent with a Fucα1–4GlcNAc assignment. The αFuc residue J appears to be fully substituted, consistent with the methylation analysis (see below). ROEs from residue K/K′ H-1 to M/M′ H-1 and 2 and from M/M′ H-1 to J-H4 suggests a Xyl*p*β1–2Gal*p*α1–4Fuc sequence. Further ROEs from N-H1 to residue J-H3 and from L-Me to J-H1 suggest that terminal Gal*p*β1–3Fuc and terminal Rha*p*α1–2Fuc linkages, respectively, complete substitution of the Fucose.

**TABLE 3:** **NMR ^1^H chemical shift assignments** The chemical shifts, given in ppm, were assigned from analysis of two-dimensional COSY and two-dimensional TOCSY spectra. The values in italics were assigned from the TOCSY spectrum alone. The axial and equatorial assignments of the Xyl H5 protons are indicated by a and e, respectively. Me denotes the methyl protons of Fuc and Rha, Ac denotes the methyl protons of GlcNac.

Label	Sugar	H1	H2	H3	H4	H5	H5′e	H6	H6′	Me	Ac
A	βGal*f*	5.09	4.13	4.23	*3.84*	*3.92*		*3.67*	*3.78*		
B	αRha	4.91	4.01	3.93	3.50	3.99				1.31	
C	αFuc	5.19	3.89	3.95	3.83	4.59				1.21	
D	βXyl	4.82	3.79	4.15	3.87	3.50a	4.19e				
E	αGal*p*	5.46	3.82	*3.86*	*3.92*	*3.98*					
F	βGal*p*	4.68	3.52	3.63	*3.94*	*3.68*		*3.91*			
G	βXyl	4.48	3.18	3.56	3.93	3.38a	4.15e				
H'	βGlcNac	4.64	3.32	*3.46*	*3.67*	*3.49*		*3.96*	*3.86*		2.10
H	αGlcNac	5.13	4.16	4.06	*3.50*	*3.93*		*3.96*	3.86		
K	βXyl	4.44	3.40	3.48	3.66	3.29a	3.96e				
K'	βXyl	4.56	3.43	3.31	3.61	3.28a	3.97e				
M	αGal*p*	5.30	4.32	4.13	*3.97*						
M'	αGal*p*	5.25	4.28	4.11							
J	αFuc	5.08	3.95	4.13	3.92	4.62				1.31	
L	αRha	?	?	3.86	3.45	3.93				1.29	
N	βGal*p*	4.71	3.73	3.54	*3.82*	*3.91*		*3.89*			

**TABLE 4:** **WIC29.26 epitope inter-residue ROEs** The chemical shifts of each ROESY cross-peak (H-1 and H-2) are given in ppm. The numbering of the ROEs corresponds to those indicated in Fig. 2. Me denotes the methyl protons of Fuc and Rha.

ROE number	H-1	Assignment			H-2	Assignment			ROE strength
		Sugar	Label	H		Sugar	Label	H	
1	5.09	Gal*f*	A	H1	3.50	Rha	B	H4	Strong
2	5.09	Gal*f*	A	H1	1.31	Rha	B	Me	Weak
3	4.91	Rha	B	H1	5.19	Fuc	C	H1	Strong
4	4.91	Rha	B	H1	3.90	Fuc	C	H2	Strong
5	4.91	Rha	B	H1	3.95	Fuc	C	H3	Weak
6	5.19	Fuc	C	H1	4.19	Xyl	D	H5e	Weak
7	5.19	Fuc	C	H1	3.87	Xyl	D	H4	Strong
8	4.59	Fuc	C	H5	3.50	Xyl	D	H5a	Medium
9	5.46	Gal*p*	E	H1	4.82	Xyl	D	H1	Weak
10	5.46	Gal*p*	E	H1	3.79	Xyl	D	H2	Medium
11	4.68	Gal*p*	F	H1	4.15	Xyl	D	H3	Medium
12	4.68	Gal*p*	F	H1	3.85	Xyl	D	H4	Medium
13	4.68	Galp	F	H1	3.79	Xyl	D	H2	Weak
14	4.82	Xyl	D	H1	3.93	Xyl	G	H4	Weak
15	4.82	Xyl	D	H1	4.15	Xyl	G*^a^*	H5e	Strong
16	4.48	Xyl	G	H1	4.06	GlcNac	H	H3	Strong
17	4.44	Xyl	K	H1	5.31	Gal*p*	M	H1	Strong
18	4.44	Xyl	K	H1	4.32	Gal*p*	M	H2	Strong
19	5.31	Gal*p*	M	H1	3.92	Fuc	J	H4	Medium
20	5.31	Gal*p*	M	H1	3.86	*^a^*			Medium
21	4.62	Fuc	J	H5	3.18	Xyl	G	H2	Medium
22	1.29	Rha	L	Me	5.08	Fuc	J	H1	Medium
23	4.71	Gal*p*	N	H1	4.13	Fuc	J	H3	Medium

*^a^* Ambiguous.

##### Methylation Linkage Analysis of the aq. HF-released Glycans

**FIGURE 3.**
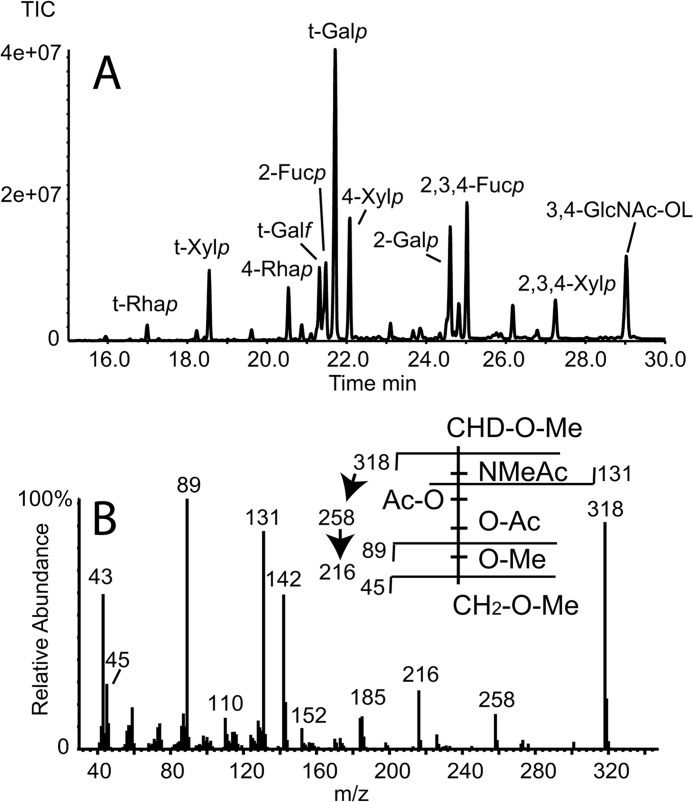
**GC-MS methylation linkage analysis of aq. HF-released glycans.** The sample recovered from NMR analysis was reduced with NaBD_4_ and converted to PMAAs for analysis by GC-MS. *A*, total ion chromatogram (*TIC*) of the PMAA derivatives of the monosaccharide constituents, annotated using descriptors that are explained in Table 5. The PMAA identities were inferred from their retention times and electron impact mass spectra. *B*, electron impact mass spectrum and spectrum assignment (*inset*) of the 3,4-disubstituted-GlcNAc-ol PMAA from *A*.

Following NMR analysis, the sample was dried and exchanged five times with H_2_O. An aliquot of the sample was reduced with NaB[^2^H]_4_ and subjected to methylation linkage analysis by GC-MS (Fig. 3*A* and Table 5). The area under each PMAA-derivative peak is not directly proportional to the relative abundance of that residue in the sample because different PMAAs produce different fragmentation patterns and, therefore, different total ion counts per mole. Nevertheless, such integrals do give an approximation of relative abundance. In general, the data are consistent with the NMR data. Thus, terminal Rha*p* (residue L), Xyl*p* (residue K), Gal*f* (residue A), and Gal*p* (residues E, F, and N) are all apparent. The low yield of the terminal Rha*p* derivative is probably due to the high volatility of this derivative. The PMAAs of the expected mono-*O*-substituted sugars are all present, *i.e.*, those corresponding to 4-*O*-substituted Rha*p* (residue B), 2-*O*-substituted Fuc*p* (residue C), 4-*O*-substituted Xyl*p* (residue E), and 2-*O*-substituted Gal*p* (residue M). Similarly, PMAAs of the predicted 2,3,4-tri-*O*-substituted Xyl*p* (residue D) and 2,3,4-tri-*O*-substituted Fuc*p* (residue J) are apparent. Traces of terminal Fuc*p* and 3,4-di-*O*-substituted Fuc*p* were also found (Table 5), suggesting heterogeneity in the substitution of Fuc*p* residues C and J by Rha*p* residues B and L, respectively. Finally, a deuteride-reduced PMAA was apparent with an electron impact spectrum consistent with a 3,4-di-*O*-substituted 1-[^2^H]-2-acetamido-2-deoxy-hexosaminitol (Fig. 3*B*). Because GlcNAc was the only *N*-acetylhexosamine apparent in the compositional analysis (Table 2) and because the NMR chemical shifts for residue H/H′ are consistent with a mutarotating reducing terminal GlcNAc residue (Table 2), we conclude that the reducing terminus of the native structure is 3,4-di-*O*-substituted GlcNAc.

**TABLE 5:** **Methylation linkage analysis of the WIC29.26 epitope**

PMAA derivative	Residue type	Relative TIC area*^a^*	Retention time
			*min*
1-[^2^H]2,3,4-Tri-*O*-methyl-1,5-di-*O*-acetyl-rhamnitol	t-Rha*p*	0.1	17.00
1-[^2^H]2,3,4-Tri-*O*-methyl-1,5-di-*O*-acetyl-fucitol	t-Fuc*p*	0.1*^b^*	18.24
1-[^2^H]2,3,4-Tri-*O*-methyl-1,5-di-*O*-acetyl-xylitol	t-Xyl*p*	0.6	18.55
1-[^2^H]2,3-Di-*O*-methyl-1,4,5-tri-*O*-acetyl-rhamnitol	4-Rha*p*	0.4	20.54
1-[^2^H]2,3,4,6-Tetra-*O*-methyl-1,5-di-*O*-acetyl-galacitol	t-Gal*f*	0.7	21.33
1-[^2^H]3,4-Di-*O*-methyl-1,2,5-tri-*O*-acetyl-fucitol	2-Fuc*p*	0.8	21.49
1-[^2^H]2,3,4,6-Tetra-*O*-methyl-1,5-di-*O*-acetyl-galacitol	t-Gal*p*	2.6	21.70
1-[^2^H]2,3-Di-*O*-methyl-1,4,5-tri-*O*-acetyl-xylitol	4-Xyl*p*	1.0	22.08
1-[^2^H]2-*O*-Methyl-1,3,4,5-tetra-*O*-acetyl-fucitol	3,4-Fuc*p*	0.1*^b^*	23.11
1-[^2^H]3,4,6-Tri-*O*-methyl-1,2,5-tri-*O*-acetyl-galacitol	2-Gal*p*	1.1	24.59
1-[^2^H]1,2,3,4,5-Penta-*O*-acetyl-fucitol	2,3,4-Fuc*p*	1.1	25.03
1-[^2^H]1,2,3,4,5-Penta-*O*-acetyl-xylitol	2,3,4-Xyl*p*	0.4	27.27
1,5,6-Tri-*O*-methyl-3,4-di-*O*-acetyl-2-*N*-methyl-acetyl-1-[^2^H]glucitol	3,4-GlcNAc-OL	1.0	28.96

*^a^* Relative total ion current (TIC) relative to that for 4-*O*-Xyl*p* = 1.0.

*^b^* Residues suggesting heterogeneity in the substitution of Fuc*p* residues C and J by Rha*p* residues B and L, respectively.

##### Electrospray Mass Spectrometric Analysis of the aq. HF-released Glycans

**FIGURE 4.**
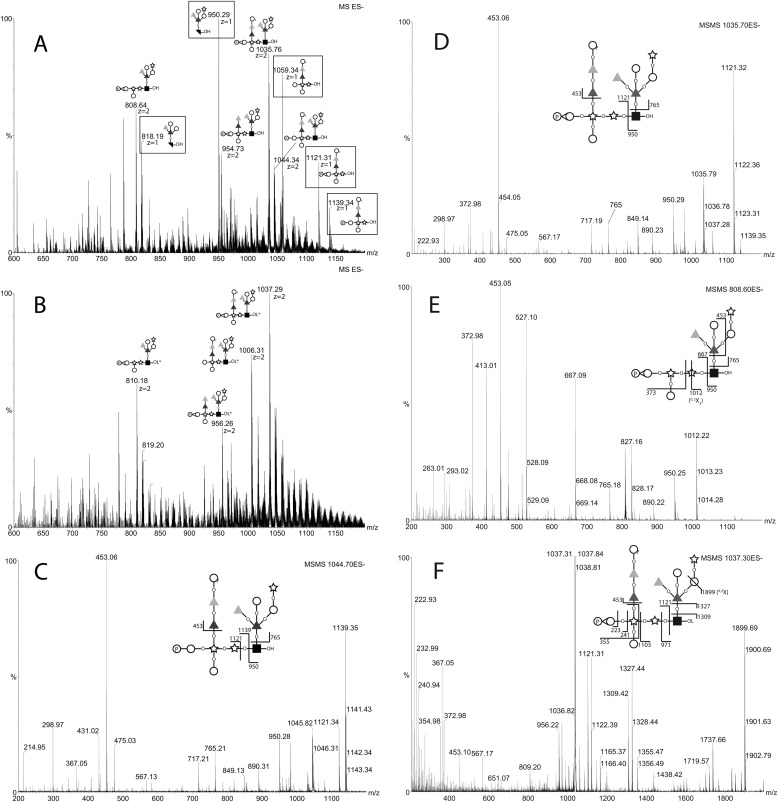
**Negative ion ES-MS and ES-MS/MS spectra of the native and reduced glycans released by aq. HF.** The glycans recovered from NMR analysis were subjected to negative ion ES-MS analysis (*A*). The major species are annotated according to their deduced compositions by accurate mass and their MS/MS spectra. The doubly charged ions (*z* = 2) appear shifted by 1.5 mass units (3 Da) after reduction with NaBD_4_ (*B*), whereas the singly charged (*z* = 1) ions (*boxed*) do not appear after reduction of the sample. The MS/MS spectra and their annotations (*insets*) of the doubly charged (*z* = 2) precursor ions in *A* at *m*/*z* 1044.34, 1035.76, and 808.64 are shown in *C*, *D*, and *E*, respectively. The MS/MS spectrum of precursor ion in *B* at *m*/*z* 1037.29 is shown in *F. Black square*, d-GlcNAc; *black bisected square*, 2,3-anhydro-d-GlcNAc; *open circle*, d-Gal*p*; *open circle* with *f* subscript, d-Gal*f*; *star*, d-Xyl*p*; *dark gray triangle*, l-Fuc*p*; *light gray triangle*, d-Rha*p. P* in a circle indicates phosphate. *–OH* indicates a reducing terminal sugar, and *–OL* indicates a deuteride-reduced terminal sugar.

**FIGURE 5.**
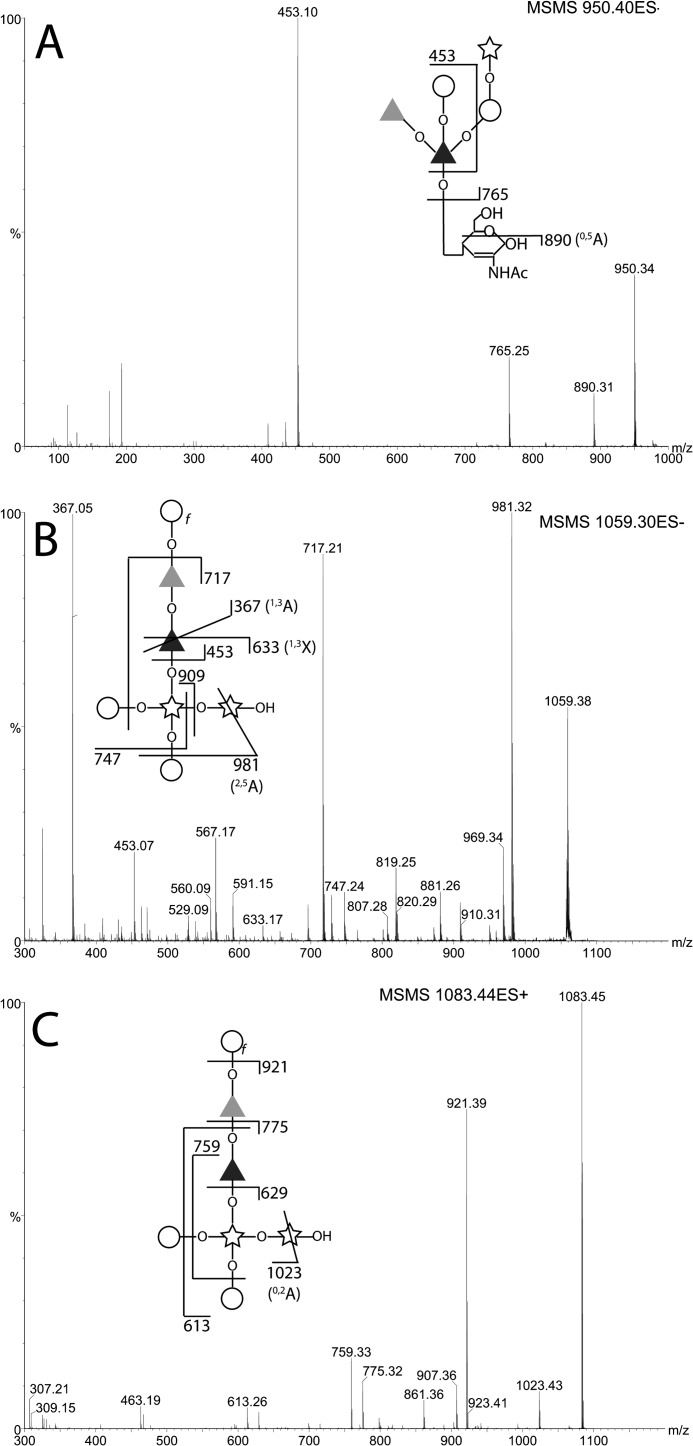
**ES-MS/MS spectra of the in-source induced fragment ions of native glycans released by aq. HF.** The singly charged (*z* = 1) [M-H]^−^ ions observed in Fig. 4*A* at *m*/*z* 950.40 and 1059.30 were isolated and subjected to CID, and their MS/MS spectra were recorded (*A* and *B*, respectively). The sample was also analyzed in positive ion mode, the [M+Na]^+^ ion at *m*/*z* 1083.44 was subjected to CID, and its MS/MS spectrum was recorded (*C*). The spectral interpretations are indicated in the *insets*. The *symbols* are as defined in the legend to Fig. 4.

The same post-NMR sample that had been dried and exchanged five times with H_2_O was analyzed by negative and positive ion ES-MS and ES-MS/MS. The negative ion spectrum showed a mixture of singly and doubly charged ions (Fig. 4*A*). The doubly charged ions at *m*/*z* 1044.8, 1035.8, 954.7, and 808.7 are related by logical losses (from *m*/*z* 1044.8) of water, water plus one hexose, and water plus one hexose and two deoxyhexoses, respectively. The *m*/*z* 1044.8 ion is consistent with being the [M-2H]^2−^ ion of the structure deduced by NMR including one phosphomonoester group (*i.e.*, composition Hex_5_dHex_4_Pent_3_HexNAc_1_P_1_). The *m*/*z* 1035.7 ion is presumably the same structure bearing a cyclic-phosphate group. Reduction of the sample with NaB[^2^H]_4_ resulted in an *m*/*z* 1.5 (equivalent to 3 Da) increase of all of the doubly charged ions, demonstrating that they all have free reducing termini, and a concomitant disappearance of the singly charged ions (Fig. 4*B*). The disappearance of the previously abundant singly charged ions at *m*/*z* 1139.3, 1121.3, 1059.3, and 950.2 upon reduction suggests that these were fragment ions formed in the source of the spectrometer because of a particularly labile linkage and that this fragmentation is stopped upon reduction. This would be consistent with a 3-*O*-linkage from the structural units responsible for these ions to the reducing terminal GlcNAc residue. Such 3-*O*-GlcNAc linkages are prone to facile β-elimination prior to, but not after, reduction. The ES-MS/MS daughter ion spectra of the *m*/*z* 1044.7, 1035.7, and 808.7 ions are also consistent with this hypothesis because they break down to give singly charged daughter ions at *m*/*z* 1139.3 and 950.3, *m*/*z* 1121.3 and 950.3, and *m*/*z* 667.1 and 950.3, respectively (Fig. 4, *C–E*). After reduction of the sample, the MS/MS spectrum of the *m*/*z* 1037.2 ion, for example, still showed the same *m*/*z* 1121.3 product ion but not the *m*/*z* 950.3 β-elimination product ion, which was replaced by an ion at *m*/*z* 971.3 that contains deuteride-reduced GlcNAc-ol. Furthermore, the product ions at *m*/*z* 1899.7, 1327.4, 1309.4, and 1103.3 are consistent with the proposed Xyl-GlcNAc-ol linkage (see Fig. 4*F*). These MS/MS spectra also show that the phosphate group belongs with the *m*/*z* 1121.3 xylobiose-containing fragment and, specifically, from the *m*/*z* 223.0, 241.0 and 335.0 ions, with a Gal residue in a Gal-Xyl sequence. Unfortunately, it is not possible to discriminate between phosphorylation of the E or the F Gal residue. The ES-MS/MS spectra of the singly charged [M-H]^−^ ions seen in Fig. 4*A* and the corresponding [M+Na]^+^ ions in positive ion mode (all arising from the in-source β-elimination reaction) are also informative and consistent with the parent structure deduced from the NMR data and methylation linkage analysis (see the annotations in Fig. 5).

**FIGURE 6.**
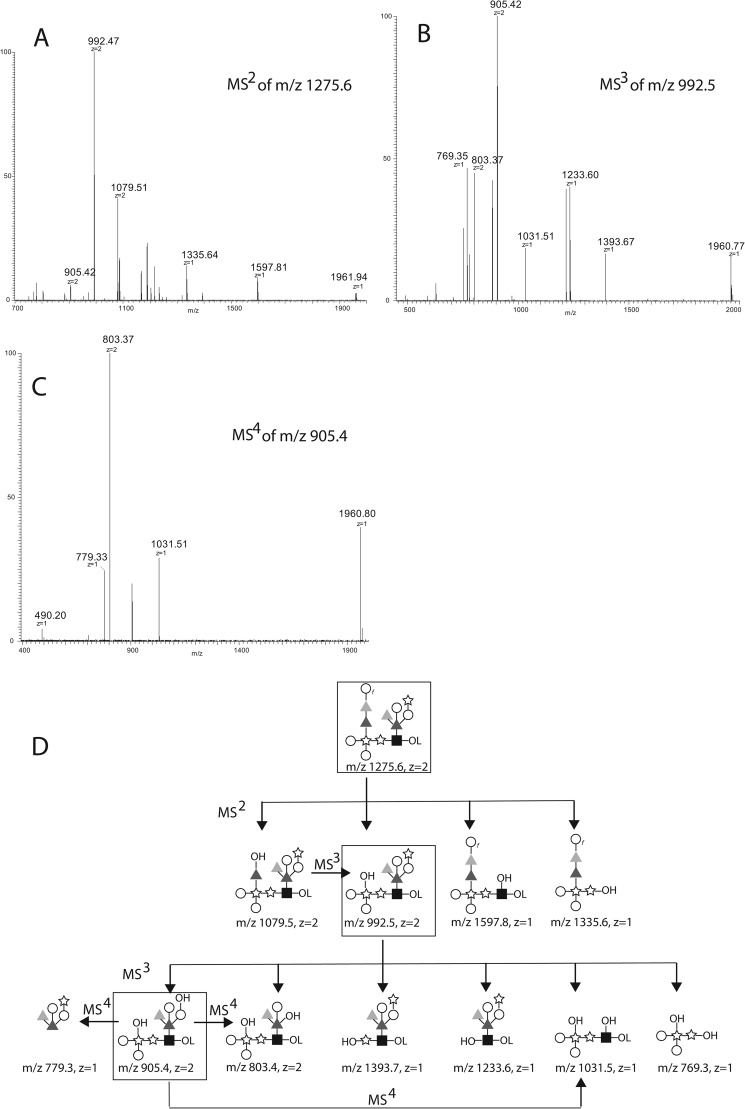
**ES-MS*^n^* analyses of reduced and permethylated derivatives of the largest glycan released by aq. HF.** *A*, the sample recovered from NMR and reduced with NaBD_4_ was permethylated and analyzed by positive ion ES-MS on an Orbitrap instrument. The [M+2Na]^2+^ ion at *m*/*z* 1275.6 was isolated and subjected to CID giving rise to the product ion (*MS^2^*) spectrum shown. *B*, the [M+2Na]^2+^ ion at *m*/*z* 992.5 from *A* was isolated and subjected to CID giving rise to the product ion (*MS^3^*) spectrum shown. *C*, the [M+2Na]^2+^ ion at *m*/*z* 905.4 from *B* was isolated and subjected to CID giving rise to the product ion (*MS^4^*) spectrum shown. *D*, the proposed fragmentation pathways corresponding to the ions observed in *A–C*. Unless otherwise noted, *z* = 2 ions are [M+2Na]^2+^ and *z* = 1 ions are [M+Na]^+^. The *symbols* are as defined in the legend to Fig. 4.

To further probe these assignments, the aq. HF-released and NaBD_4_-reduced glycans were permethylated and reanalyzed by positive ion ES-MS using a Thermo Orbitrap mass spectrometer. A doubly charged ion at *m*/*z* 1275.6 was observed, corresponding to the [M+2Na]^2+^ ion of deuteride-reduced and permethylated Hex_5_dHex_4_Pent_3_HexNAc_1_ glycan (*i.e.*, a glycan of the same composition as the *m*/*z* 1044.7 and 1035.7 native glycan species described above, but without a phosphate group). The *m*/*z* 1275.6 deuteride-reduced and permethylated [Hex_5_dHex_4_Pent_3_HexNAc_1_ + 2Na]^2+^ ion was isolated and subjected to collision-induced dissociation (CID), and the MS/MS spectrum is shown in Fig. 6*A*. The *m*/*z* 1079.5 product ion was then isolated and subjected to CID, and the MS/MS/MS spectrum showed a single major product ion at *m*/*z* 992.4. We therefore isolated and performed MS/MS/MS on the *m*/*z* 992.5 ion from the MS/MS experiment, and this produced the spectrum shown in Fig. 6*B* from which the *m*/*z* 905.4 ion was isolated and subjected to CID to produce the MS/MS/MS/MS spectrum shown in Fig. 6*C*. Our interpretation of these spectra is shown in Fig. 6*D*.

## DISCUSSION

**FIGURE 7.**
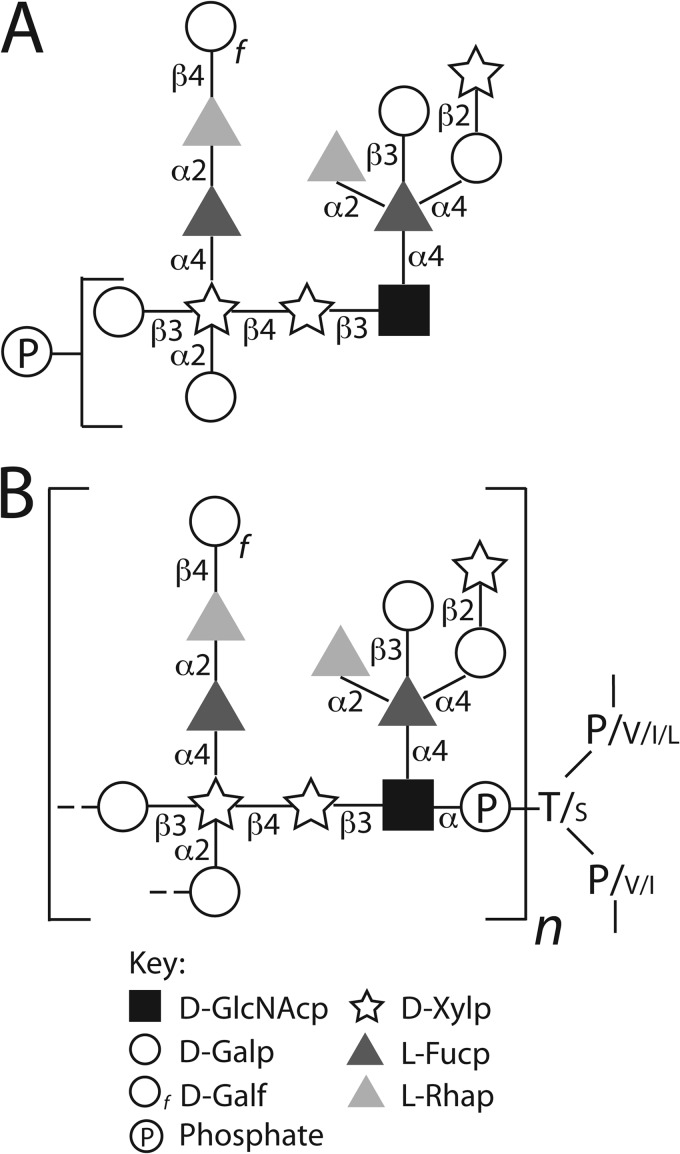
**The proposed fine structure of the WIC29.26 phosphosaccharide epitope found on the gp72 glycoprotein of *T. cruzi* epimastigotes.** *A*, the chemical structure of the largest phosphosaccharide structure released from gp72 Pronase glycopeptides by partial aq. HF dephosphorylation. The precise location of the phosphate group (found in both phosphomonoester and cyclic phosphodiester form) is unknown but is linked to one or other of the two terminal Gal*p* residues, as indicated. The heterogeneity of the structures observed by ES-MS and NMR suggests that not every phosphosaccharide unit is complete. *B*, proposed arrangement of the phosphosaccharide units on gp72. The proposed repeating pattern of *n* phosphosacchride units (with putative GlcNAcα1-P-Gal inter-repeat linkages) is based on the ratio of phosphosacchride units to potential phosphoglycosylation sites (30:18), suggested by compositional analyses of gp72 (13, 14) and its amino acid sequence (15), and is supported by the position of the phosphate group shown in *A*. The phosphodiester linkage to Thr (*T*) and Ser (*S*) is based on Ref. 11, and the sizes of the amino acid codes reflect their relative frequencies in the amino acid sequences of the putative gp72 phosphoglycosylation sites (15). The *symbols* are as defined in the legend to Fig. 4.

The structure of the WIC29.26 phosphosaccharide epitope and its likely linkage to the gp72 polypeptide (11) is presented in Fig. 7. We have assumed, based on the identification of phosphothreonine and some phosphoserine in partial acid hydolysates of ^32^P-labeled gp72 (11), that the reducing terminal GlcNAc residue is in phosphodiester linkage to Thr/Ser residues in the gp72 polypeptide. We further assume that this will be in α-anomeric linkage, with the GlcNAcα1-*P* moiety coming from UDP-αGlcNAc. There is a precedent for such an arrangement (GlcNAcα1-*O*-Ser) in *D. discoidium* proteinase I (10, 27).

Unfortunately, we were unable to obtain mass spectrometric data on the intact Pronase glycopeptides, which might have resolved whether the phosphosaccharide units form repeating structures (as seen in the *Leishmania* proteophosphoglycans (7)) or are present as single units. However, amino acid and carbohydrate composition analyses have shown that gp72 is ∼50% carbohydrate, by weight (13, 14). Because the predicted molecular mass of mature gp72 polypeptide is 59.4 kDa and the mass of a complete phosphosaccharide unit is 2093.8 Da, we estimate that each gp72 molecule carries, on average, around 30 phosphosaccharide units. The gp72 predicted amino acid sequence (15) contains a Thr- and Pro-rich domain (PTVTPTPPLTPTPTPEVTPTPTVTPTPTPEVTPTPPVTPSPTITI) that has 17 Thr sites and one Ser site predicted to be potential *O-*glycosylation sites by the program NetOGlyc. Assuming these also represent the potential sites of phosphoglycosylation in gp72, it seems likely that at least some gp72 phosphoglycosylation sites are occupied by repeating, rather than single, phosphosaccharide units. Indeed, the presence of phosphate on a significant proportion of the terminal Gal*p* residues of the released units (Fig. 4) is suggestive of the presence of phosphosaccharide repeats.

The structure presented here is a significant advance on the partial structure presented in Ref. 11. First, it discriminates Gal*p* from Gal*f* (hexose) residues and Fuc from Rha (deoxyhexose) residues and provides absolute configuration (d or l) and anomeric (α or β) information. It also identifies GlcNAc as a structural component of the WIC29.26 carbohydrate epitope for the first time. Previously, GlcNAc had been observed as a compositional component in the epitope from Y strain gp72 (13) but was not found in the Y strain partial epitope structure described in Ref. 11. The 3-*O*-substitution of the reducing terminal GlcNAc residue described here (which is prone to alkali-induced β-elimination or “peeling” reactions) probably explains why this sugar previously “disappeared” in structural analyses. Thus, in Ref. 11, mild acid-released oligosaccharides were reduced in strong base (100 mm NaOH) containing a low concentration (36 mm) of NaB^3^H_4_ reductant before further work-up. This will have allowed the base-catalyzed β-elimination and reduction of the (base-stable) xylobiose-containing component, partially characterized in that paper but destruction of the reducing-terminal GlcNAc residue.

The principal glycoprotein that contains the WIC29.26 carbohydrate epitope is the relatively low abundance gp72 molecule that localizes to the flagellar attachment zone (16, 29) and is essential for flagellar attachment to the cell body (17, 18). However, what role this complex, highly immunogenic, phosphosaccharide structure plays in this function of gp72 is unknown. To date, the genes for the sugar-phosphate transferases responsible for initiating kinetoplastid P-linked glycans and for elaborating them have not been reported. Thus, it is not yet possible to probe the function of the P-linked glycan chains independently of the glycoprotein polypeptide components through genetic procedures. Two homologous glycoproteins in *T. brucei* (Fla1 and Fla2) are also involved in flagellar adhesion in that parasite (30), but again, the role that carbohydrate plays in this process is unknown. In any case, the carbohydrate chains of Fla1 and Fla2 will presumably be different from those of *T. cruzi* gp72, because *T. brucei* does not have the capacity to synthesize Xyl, Rha, or Gal*f* (28).

Finally, it has been shown that the binding of WIC29.26 mAb to *T. cruzi* epimastigotes prevents their subsequent differentiation to nondividing (human infectious) metacylic trypomastigote forms (20). This raises the possibility that an analogous interaction between the gp72 on the surface of *T. cruzi* epimastigotes and a putative lectin in the digestive tract of the insect vector might play a role in establishing and maintaining the infection in the vector, with escape from the lectin interaction permitting differentiation into the metacyclic trypomastigote form. This hypothesis has similarities to the well described interactions for the related leishmania parasites. These organisms multiply as procyclic-promastigote forms in the mid-gut of the sandfly vector, where they bind to insect gut wall lectins through their lipophosphoglycan surface molecules. Changes in lipophosphoglycan structure, as the leishmania parasites mature into nondividing and human-infectious metacyclic-promastigote forms, result in their detachment and migration to the mouthparts for subsequent transmission (31–34). The analogous migration of *T. cruzi* metacyclic trypomastigotes for transmission to the host is to the hind-gut and rectum of the insect vector rather than the mouthparts, but otherwise the scenario could be quite similar.

## Footnotes

4 The monosaccharide abbreviations used are: Ara, arabinose; Fuc, fucose; Rha, rhamnose; Xyl, xylose. Their absolute configurations are defined by d and l, and their ring configurations, pyranose or furanose, are indicated by *p* and *f*, respectively.

## Footnotes

3 The abbreviations used are:

P-linked: phosphate-linked
aq. HF: aqueous hydrofluoric acid
COSY: correlation spectroscopy
TOCSY: total correlation spectroscopy
ROE: rotating frame nuclear Overhauser effect
ROESY: ROE spectroscopy
PMAA: partially methylated alditol acetate
ES: Electrospray
CID: collision-induced dissociation.

## References

[1] Acosta-Serrano A. Almeida I. C. Freitas-Junior L. H. Yoshida N. Schenkman S. (2001) The mucin-like glycoprotein super-family of *Trypanosoma cruzi.* Structure and biological roles. Mol. Biochem. Parasitol. 114, 143–150 11378194 10.1016/s0166-6851(01)00245-6

[2] Buscaglia C. A. Campo V. A. Frasch A. C. Di Noia J. M. (2006) *Trypanosoma cruzi* surface mucins. Host-dependent coat diversity. Nat. Rev. Microbiol. 4, 229–236 16489349 10.1038/nrmicro1351

[3] Giorgi M. E. de Lederkremer R. M. (2011) Trans-sialidase and mucins of *Trypanosoma cruzi.* An important interplay for the parasite. Carbohydr. Res. 346, 1389–1393 21645882 10.1016/j.carres.2011.04.006

[4] Mendonça-Previato L. Todeschini A. R. Heise N. Previato J. O. (2005) Protozoan parasite-specific carbohydrate structures. Curr. Opin. Struct. Biol. 15, 499–505 16154349 10.1016/j.sbi.2005.08.011

[5] Soares R. P. Torrecilhas A. C. Assis R. R. Rocha M. N. Moura e Castro F. A. Freitas G. F. Murta S. M. Santos S. L. Marques A. F. Almeida I. C. Romanha A. J. (2012) Intraspecies variation in *Trypanosoma cruzi* GPI-mucins. Biological activities and differential expression of α-galactosyl residues. Am. J. Trop. Med. Hyg. 87, 87–96 22764297 10.4269/ajtmh.2012.12-0015 PMC3391063

[6] Macrae J. I. Acosta-Serrano A. Morrice N. A. Mehlert A. Ferguson M. A. (2005) Structural characterization of NETNES, a novel glycoconjugate in *Trypanosoma cruzi* epimastigotes. J. Biol. Chem. 280, 12201–12211 15649890 10.1074/jbc.M412939200

[7] Ilg T. (2000) Proteophosphoglycans of *Leishmania*. Parasitol. Today 16, 489–497 11063860 10.1016/s0169-4758(00)01791-9

[8] Thomson L. M. Lamont D. J. Mehlert A. Barry J. D. Ferguson M. A. (2002) Partial structure of glutamic acid and alanine-rich protein, a major surface glycoprotein of the insect stages of *Trypanosoma congolense*. J. Biol. Chem. 277, 48899–48904 12368279 10.1074/jbc.M208942200

[9] Haynes P. A. (1998) Phosphoglycosylation. A new structural class of glycosylation? Glycobiology 8, 1–5 9451009 10.1093/glycob/8.1.1

[10] Mreyen M. Champion A. Srinivasan S. Karuso P. Williams K. L. Packer N. H. (2000) Multiple O-glycoforms on the spore coat protein SP96 in *Dictyostelium discoideum.* Fuc(α1–3)GlcNAc-α-1-P-Ser is the major modification. J. Biol. Chem. 275, 12164–12174 10766852 10.1074/jbc.275.16.12164

[11] Haynes P. A. Ferguson M. A. Cross G. A. (1996) Structural characterization of novel oligosaccharides of cell-surface glycoproteins of *Trypanosoma cruzi*. Glycobiology 6, 869–878 9023550 10.1093/glycob/6.8.869

[12] Snary D. Ferguson M. A. Scott M. T. Allen A. K. (1981) Cell surface antigens of *Trypanosoma cruzi.* Use of monoclonal antibodies to identify and isolate an epimastigote specific glycoprotein. Mol. Biochem. Parasitol. 3, 343–356 6795502 10.1016/0166-6851(81)90035-9

[13] Ferguson M. A. Allen A. K. Snary D. (1983) Studies on the structure of a phosphoglycoprotein from the parasitic protozoan *Trypanosoma cruzi*. Biochem. J. 213, 313–319 6351838 10.1042/bj2130313 PMC1152130

[14] Ferguson M. A. Snary D. Allen A. K. (1985) Comparative compositions of cell surface glycoconjugates isolated from *Trypanosoma cruzi* epimastigotes. Biochim. Biophys. Acta 842, 39–44 3899181 10.1016/0304-4165(85)90290-9

[15] Cooper R. Inverso J. A. Espinosa M. Nogueira N. Cross G. A. (1991) Characterization of a candidate gene for GP72, an insect stage-specific antigen of *Trypanosoma cruzi*. Mol. Biochem. Parasitol. 49, 45–59 1840630 10.1016/0166-6851(91)90129-t

[16] Haynes P. A. Russell D. G. Cross G. A. (1996) Subcellular localization of *Trypanosoma cruzi* glycoprotein Gp72. J. Cell Sci. 109, 2979–2988 9004033 10.1242/jcs.109.13.2979

[17] Cooper R. de Jesus A. R. Cross G. A. (1993) Deletion of an immunodominant *Trypanosoma cruzi* surface glycoprotein disrupts flagellum-cell adhesion. J. Cell Biol. 122, 149–156 8314840 10.1083/jcb.122.1.149 PMC2119612

[18] de Jesus A. R. Cooper R. Espinosa M. Gomes J. E. Garcia E. S. Paul S. Cross G. A. (1993) Gene deletion suggests a role for *Trypanosoma cruzi* surface glycoprotein GP72 in the insect and mammalian stages of the life cycle. J. Cell Sci. 106, 1023–1033 8126090 10.1242/jcs.106.4.1023

[19] Basombrío M. A. Gómez L. Padilla A. M. Ciaccio M. Nozaki T. Cross G. A. (2002) Targeted deletion of the gp72 gene decreases the infectivity of *Trypanosoma cruzi* for mice and insect vectors. J. Parasitol. 88, 489–493 12099416 10.1645/0022-3395(2002)088[0489:TDOTGG]2.0.CO;2

[20] Sher A. Snary D. (1982) Specific inhibition of the morphogenesis of *Trypanosoma cruzi* by a monoclonal antibody. Nature 300, 639–640 6755270 10.1038/300639a0

[21] Ferguson M. A. J. (1994) In Glycobiology: A Practical Approach (Fukuda M. Kobata A., eds) pp. 349–383, IRL Oxford University Press, Oxford

[22] Gerwig G. J. Kamerling J. P. Vliegenthart J. F. (1979) Determination of the absolute configuration of mono-saccharides in complex carbohydrates by capillary G.L.C. Carbohydr. Res. 77, 10–17 519653 10.1016/s0008-6215(00)83788-x

[23] McConville M. J. Thomas-Oates J. E. Ferguson M. A. Homans S. W. (1990) Structure of the lipophosphoglycan from *Leishmania major*. J. Biol. Chem. 265, 19611–19623 2246247

[24] Schneider P. McConville M. J. Ferguson M. A. (1994) Characterization of GDP-α-d-arabinopyranose, the precursor of d-Arap in *Leishmania major* lipophosphoglycan. J. Biol. Chem. 269, 18332–18337 8034578

[25] Gorin P. A. Previato J. O. Mendonça-Previato L. Travassos L. R. (1979) Structure of the d-mannan and d-arabino-d-galactan in *Crithidia fasciculata.* Changes in proportion with age of culture. J. Protozool. 26, 473–478 536936 10.1111/j.1550-7408.1979.tb04656.x

[26] Schneider P. Treumann A. Milne K. G. McConville M. J. Zitzmann N. Ferguson M. A. (1996) Structural studies on a lipoarabinogalactan of *Crithidia fasciculata*. Biochem. J. 313, 963–971 8611182 10.1042/bj3130963 PMC1217005

[27] Gustafson G. L. Milner L. A. (1980) Occurrence of *N*-acetylglucosamine-1-phosphate in proteinase I from *Dictyostelium discoideum*. J. Biol. Chem. 255, 7208–7210 6993483

[28] Turnock D. C. Ferguson M. A. (2007) Sugar nucleotide pools of *Trypanosoma brucei*, *Trypanosoma cruzi*, and *Leishmania major*. Eukaryotic Cell 6, 1450–1463 17557881 10.1128/EC.00175-07 PMC1951125

[29] Rocha G. M. Brandão B. A. Mortara R. A. Attias M. de Souza W. Carvalho T. M. (2006) The flagellar attachment zone of *Trypanosoma cruzi* epimastigote forms. J. Struct. Biol. 154, 89–99 16414276 10.1016/j.jsb.2005.11.008

[30] LaCount D. J. Barrett B. Donelson J. E. (2002) *Trypanosoma brucei* FLA1 is required for flagellum attachment and cytokinesis. J. Biol. Chem. 277, 17580–17588 11877446 10.1074/jbc.M200873200

[31] Dobson D. E. Kamhawi S. Lawyer P. Turco S. J. Beverley S. M. Sacks D. L. (2010) *Leishmania major* survival in selective *Phlebotomus papatasi* sand fly vector requires a specific SCG-encoded lipophosphoglycan galactosylation pattern. PLoS Pathog. 6, e1001185 21085609 10.1371/journal.ppat.1001185 PMC2978724

[32] Kamhawi S. Modi G. B. Pimenta P. F. Rowton E. Sacks D. L. (2000) The vectorial competence of *Phlebotomus sergenti* is specific for *Leishmania tropica* and is controlled by species-specific, lipophosphoglycan-mediated midgut attachment. Parasitology 121, 25–33 11085222 10.1017/s0031182099006125

[33] Kamhawi S. Ramalho-Ortigao M. Pham V. M. Kumar S. Lawyer P. G. Turco S. J. Barillas-Mury C. Sacks D. L. Valenzuela J. G. (2004) A role for insect galectins in parasite survival. Cell 119, 329–341 15543683 10.1016/j.cell.2004.10.009

[34] McConville M. J. Turco S. J. Ferguson M. A. Sacks D. L. (1992) Developmental modification of lipophosphoglycan during the differentiation of *Leishmania major* promastigotes to an infectious stage. EMBO J. 11, 3593–3600 1396559 10.1002/j.1460-2075.1992.tb05443.x PMC556818

